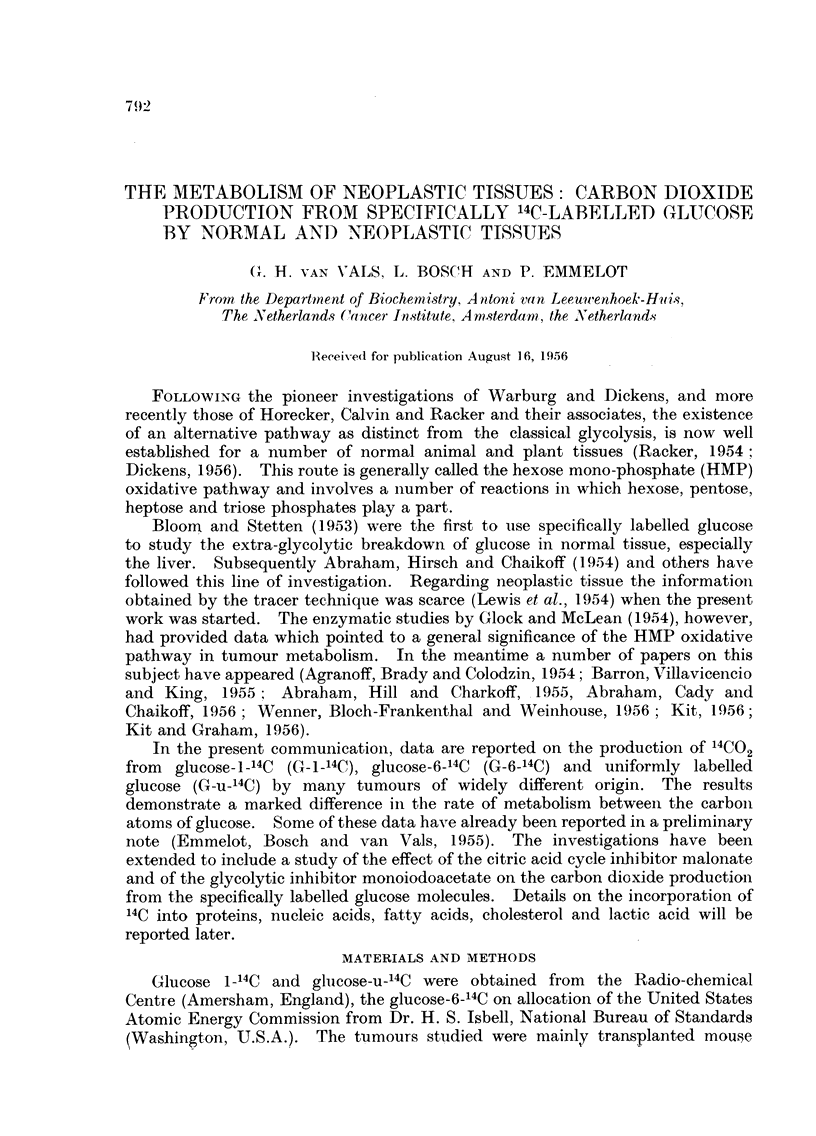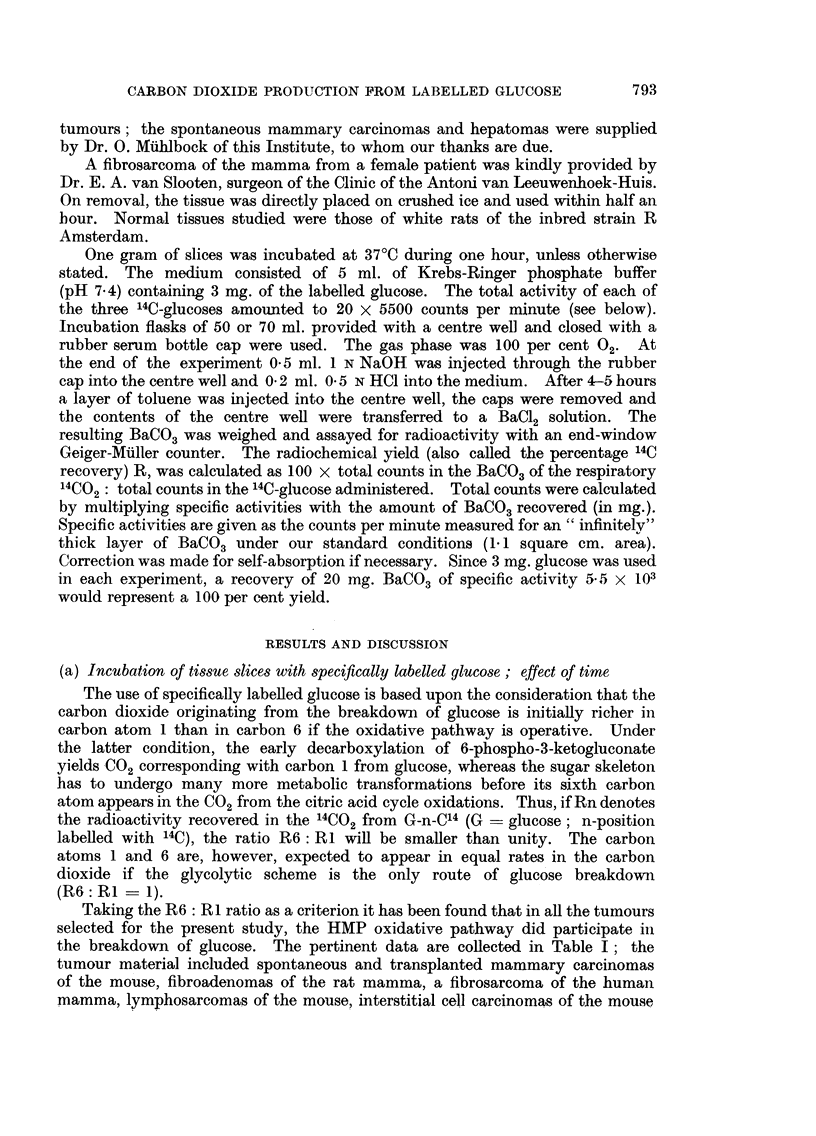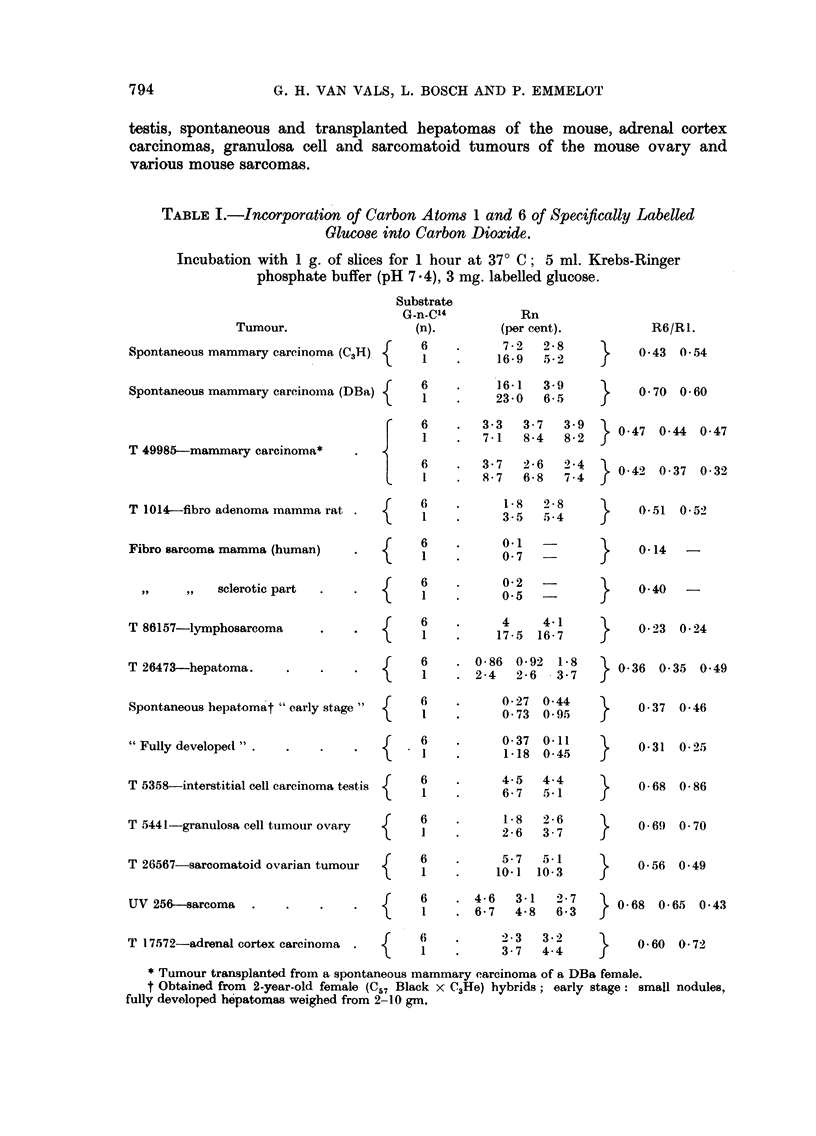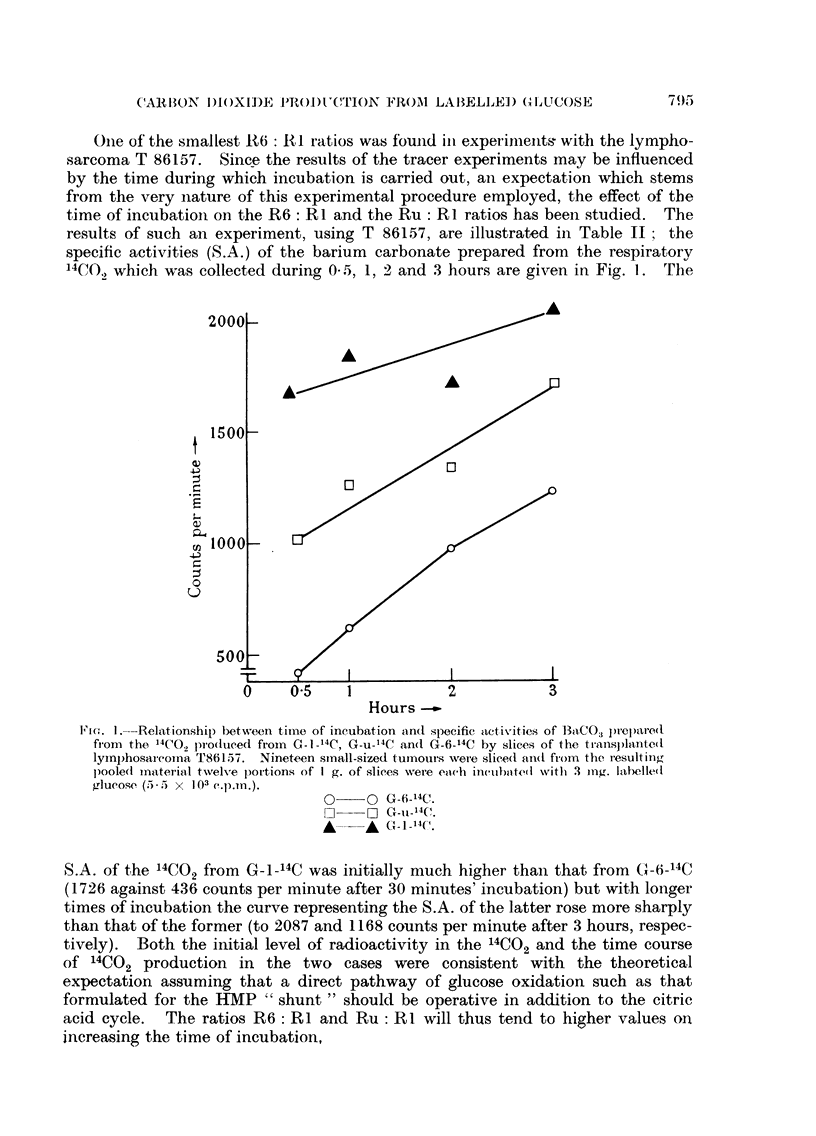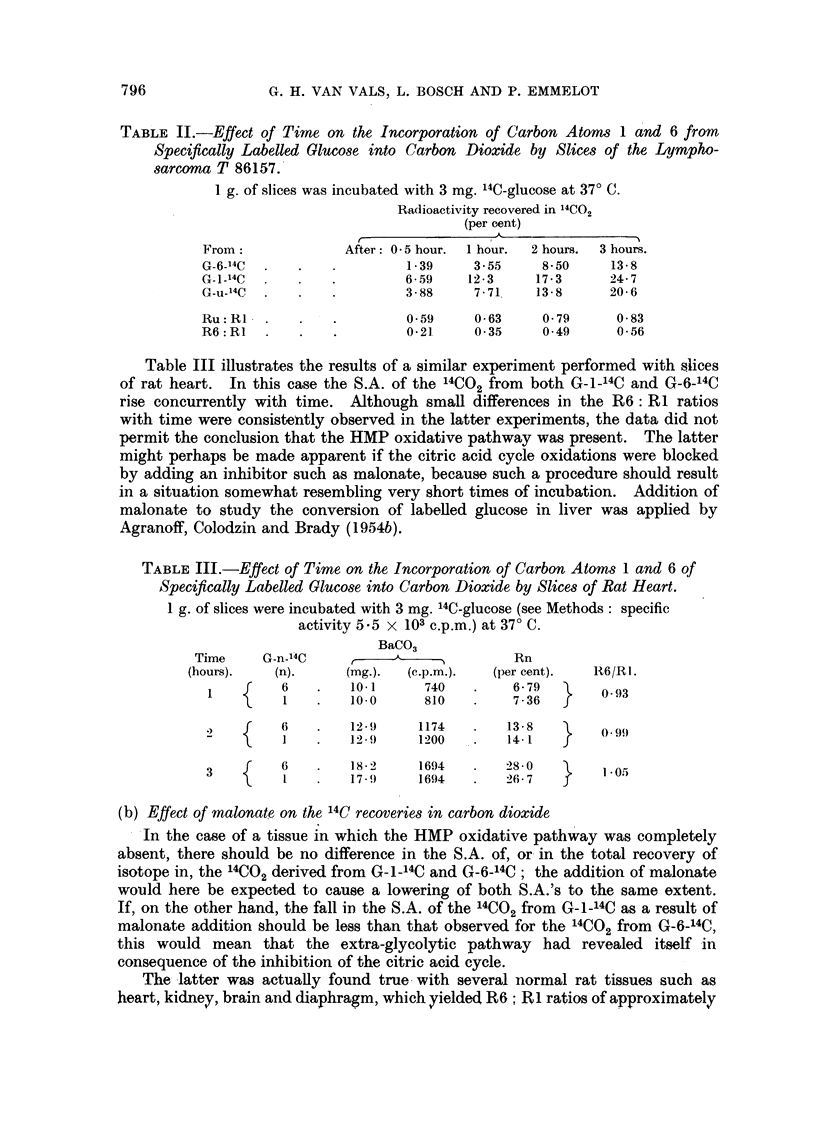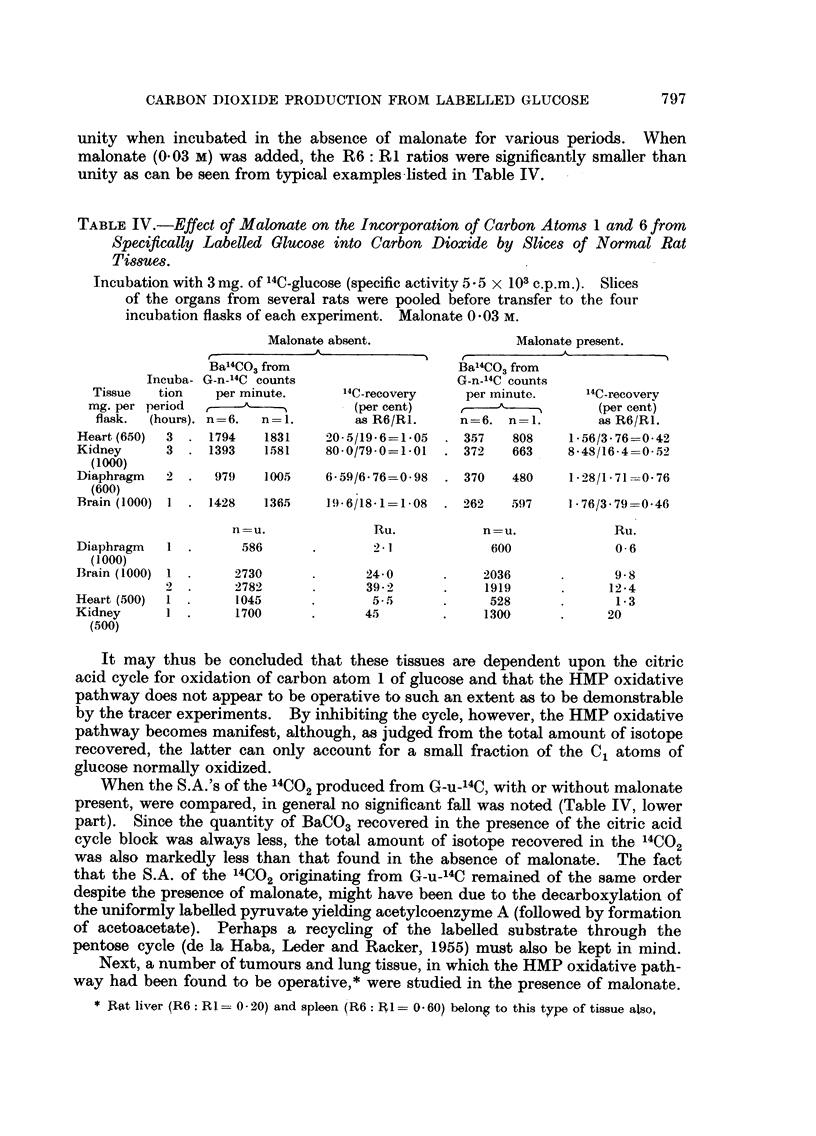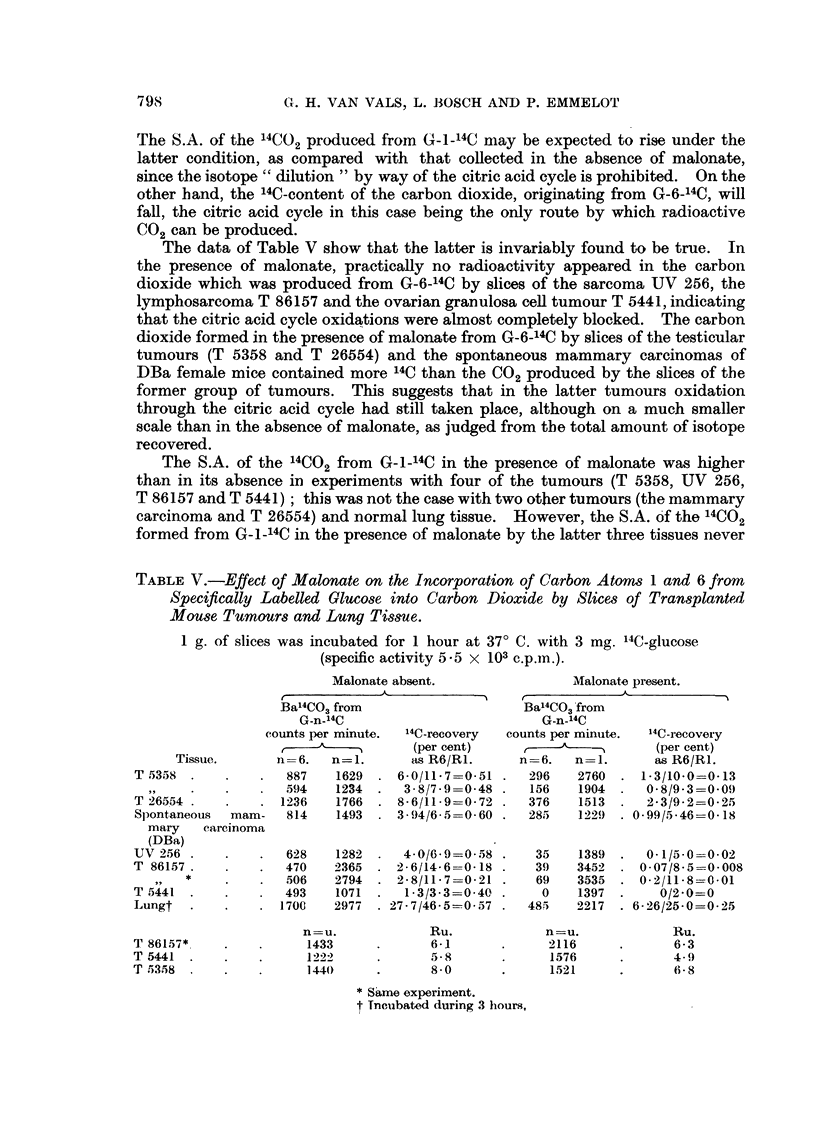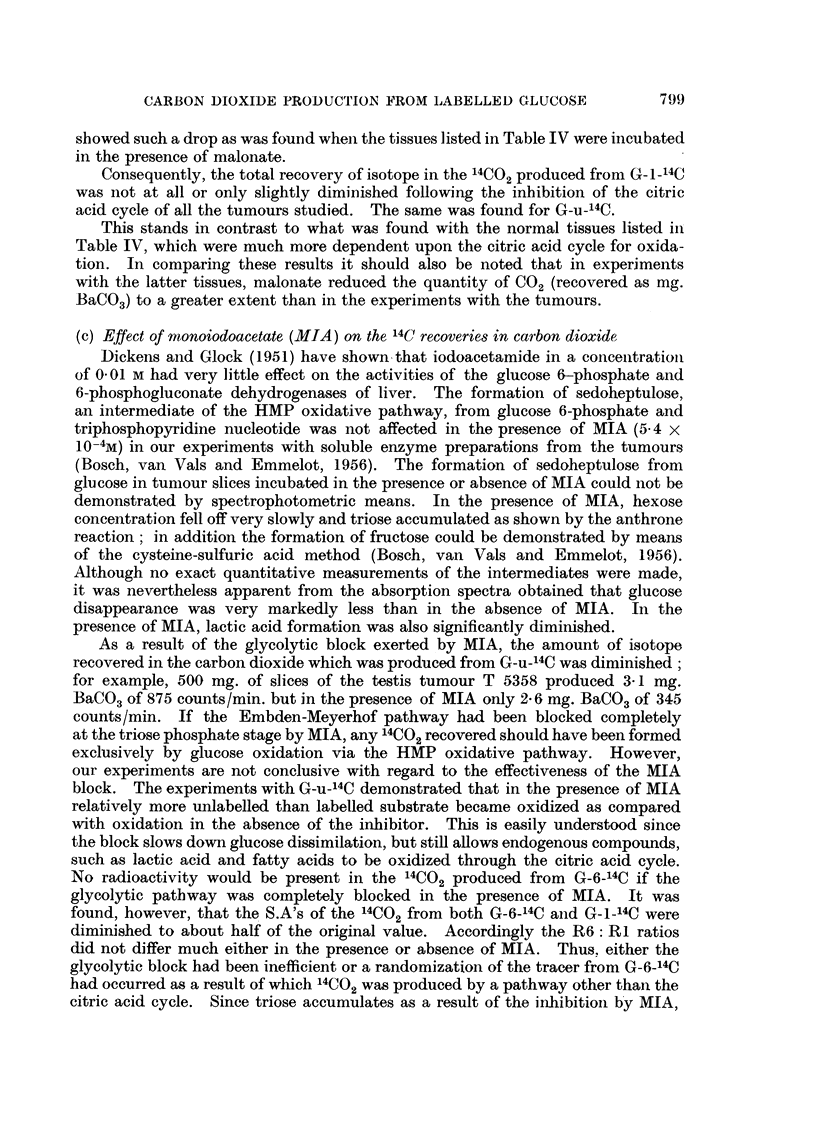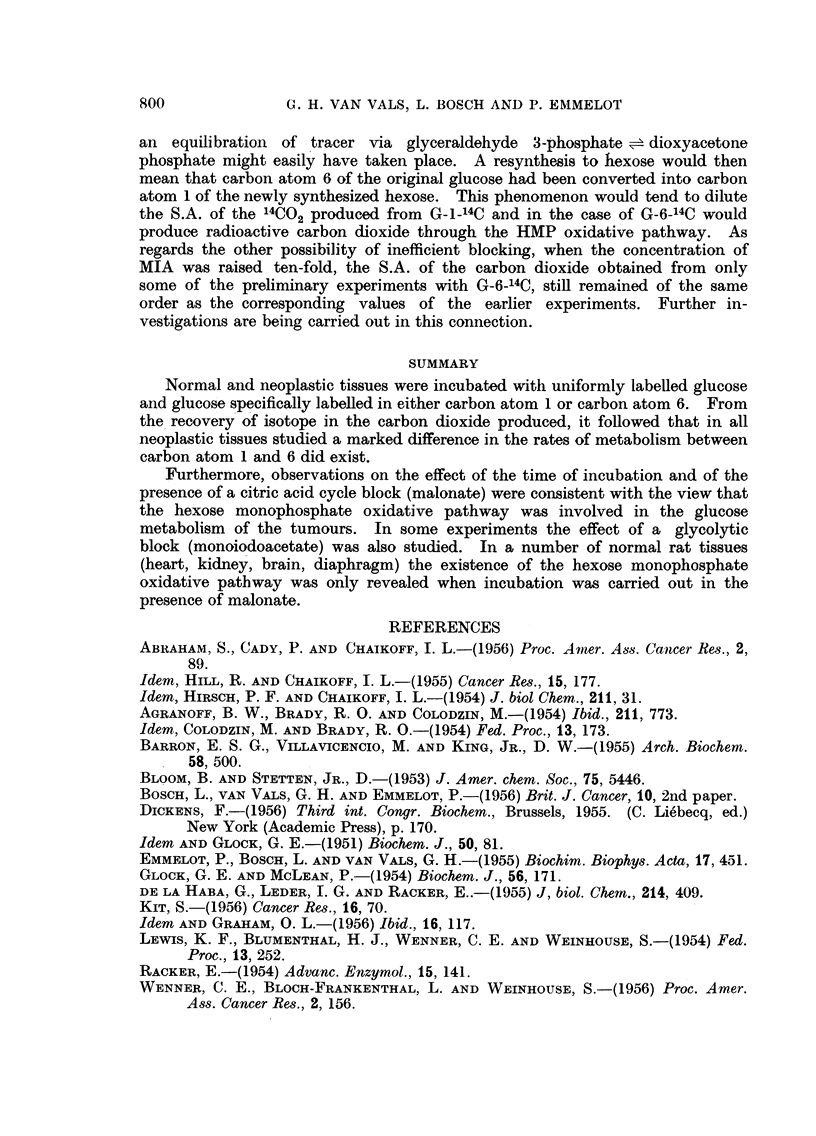# The Metabolism of Neoplastic Tissues: Carbon Dioxide Production from Specifically ^14^C-Labelled Glucose by Normal and Neoplastic Tissues

**DOI:** 10.1038/bjc.1956.96

**Published:** 1956-12

**Authors:** G. H. van Vals, L. Bosch, P. Emmelot


### THE METABOLISM OF NEOPLASTIC TISSUES: CARBON DIOXIDE PRODUCTION FROM SPECIFICALLY $^{14}C$-LABELLED GLUCOSE BY NORMAL AND NEOPLASTIC TISSUES

G. H. VAN VALS, L. BOSCH AND P. EMMELOT

*From the Department of Biochemistry, Antoni van Leeuwenhoek-Huis, The Netherlands Cancer Institute, Amsterdam, the Netherlands*

Received for publication August 16, 1956

FOLLOWING the pioneer investigations of Warburg and Dickens, and more recently those of Horecker, Calvin and Racker and their associates, the existence of an alternative pathway as distinct from the classical glycolysis, is now well established for a number of normal animal and plant tissues (Racker, 1954; Dickens, 1956). This route is generally called the hexose mono-phosphate (HMP) oxidative pathway and involves a number of reactions in which hexose, pentose, heptose and triose phosphates play a part.

Bloom and Stetten (1953) were the first to use specifically labelled glucose to study the extra-glycolytic breakdown of glucose in normal tissue, especially the liver. Subsequently Abraham, Hirsch and Chaikoff (1954) and others have followed this line of investigation. Regarding neoplastic tissue the information obtained by the tracer technique was scarce (Lewis *et al.*, 1954) when the present work was started. The enzymatic studies by Glock and McLean (1954), however, had provided data which pointed to a general significance of the HMP oxidative pathway in tumour metabolism. In the meantime a number of papers on this subject have appeared (Agranoff, Brady and Colodzin, 1954; Barron, Villavicencio and King, 1955; Abraham, Hill and Charkoff, 1955, Abraham, Cady and Chaikoff, 1956; Wenner, Bloch-Frankenthal and Weinhouse, 1956; Kit, 1956; Kit and Graham, 1956).

In the present communication, data are reported on the production of $^{14}CO_2$ from glucose-1-$^{14}C$ (G-1-$^{14}C$), glucose-6-$^{14}C$ (G-6-$^{14}C$) and uniformly labelled glucose (G-u-$^{14}C$) by many tumours of widely different origin. The results demonstrate a marked difference in the rate of metabolism between the carbon atoms of glucose. Some of these data have already been reported in a preliminary note (Emmelot, Bosch and van Vals, 1955). The investigations have been extended to include a study of the effect of the citric acid cycle inhibitor malonate and of the glycolytic inhibitor monoiodoacetate on the carbon dioxide production from the specifically labelled glucose molecules. Details on the incorporation of $^{14}C$ into proteins, nucleic acids, fatty acids, cholesterol and lactic acid will be reported later.

### MATERIALS AND METHODS

Glucose 1-$^{14}C$ and glucose-u-$^{14}C$ were obtained from the Radio-chemical Centre (Amersham, England), the glucose-6-$^{14}C$ on allocation of the United States Atomic Energy Commission from Dr. H. S. Isbell, National Bureau of Standards (Washington, U.S.A.). The tumours studied were mainly transplanted mouse tumours ; the spontaneous mammary carcinomas and hepatomas were supplied by Dr. O. Mühlbock of this Institute, to whom our thanks are due.

A fibrosarcoma of the mamma from a female patient was kindly provided by Dr. E. A. van Slooten, surgeon of the Clinic of the Antoni van Leeuwenhoek-Huis. On removal, the tissue was directly placed on crushed ice and used within half an hour. Normal tissues studied were those of white rats of the inbred strain R Amsterdam.

One gram of slices was incubated at 37°C during one hour, unless otherwise stated. The medium consisted of 5 ml. of Krebs-Ringer phosphate buffer (pH 7·4) containing 3 mg. of the labelled glucose. The total activity of each of the three $^{14}C$-glucoses amounted to 20 × 5500 counts per minute (see below). Incubation flasks of 50 or 70 ml. provided with a centre well and closed with a rubber serum bottle cap were used. The gas phase was 100 per cent $O_2$. At the end of the experiment 0·5 ml. 1 N NaOH was injected through the rubber cap into the centre well and 0·2 ml. 0·5 N HCl into the medium. After 4–5 hours a layer of toluene was injected into the centre well, the caps were removed and the contents of the centre well were transferred to a $BaCl_2$ solution. The resulting $BaCO_3$ was weighed and assayed for radioactivity with an end-window Geiger-Müller counter. The radiochemical yield (also called the percentage $^{14}C$ recovery) R, was calculated as 100 × total counts in the $BaCO_3$ of the respiratory $^{14}CO_2$ : total counts in the $^{14}C$-glucose administered. Total counts were calculated by multiplying specific activities with the amount of $BaCO_3$ recovered (in mg.). Specific activities are given as the counts per minute measured for an " infinitely" thick layer of $BaCO_3$ under our standard conditions (1·1 square cm. area). Correction was made for self-absorption if necessary. Since 3 mg. glucose was used in each experiment, a recovery of 20 mg. $BaCO_3$ of specific activity $5{\cdot}5 \times 10^3$ would represent a 100 per cent yield.

### RESULTS AND DISCUSSION

### (a) *Incubation of tissue slices with specifically labelled glucose ; effect of time*

The use of specifically labelled glucose is based upon the consideration that the carbon dioxide originating from the breakdown of glucose is initially richer in carbon atom 1 than in carbon 6 if the oxidative pathway is operative. Under the latter condition, the early decarboxylation of 6-phospho-3-ketogluconate yields $CO_2$ corresponding with carbon 1 from glucose, whereas the sugar skeleton has to undergo many more metabolic transformations before its sixth carbon atom appears in the $CO_2$ from the citric acid cycle oxidations. Thus, if Rn denotes the radioactivity recovered in the $^{14}CO_2$ from G-n-$C^{14}$ (G = glucose ; n-position labelled with $^{14}C$), the ratio R6 : R1 will be smaller than unity. The carbon atoms 1 and 6 are, however, expected to appear in equal rates in the carbon dioxide if the glycolytic scheme is the only route of glucose breakdown (R6 : R1 = 1).

Taking the R6 : R1 ratio as a criterion it has been found that in all the tumours selected for the present study, the HMP oxidative pathway did participate in the breakdown of glucose. The pertinent data are collected in Table I ; the tumour material included spontaneous and transplanted mammary carcinomas of the mouse, fibroadenomas of the rat mamma, a fibrosarcoma of the human mamma, lymphosarcomas of the mouse, interstitial cell carcinomas of the mouse

**testis, spontaneous and transplanted hepatomas of the mouse, adrenal cortex carcinomas, granulosa cell and sarcomatoid tumours of the mouse ovary and various mouse sarcomas.**

TABLE I.—*Incorporation of Carbon Atoms 1 and 6 of Specifically Labelled Glucose into Carbon Dioxide.*

Incubation with 1 g. of slices for 1 hour at 37° C ; 5 ml. Krebs-Ringer phosphate buffer (pH 7·4), 3 mg. labelled glucose.

| Tumour. | Substrate G-n-$C^{14}$ (n). | Rn (per cent). | | | R6/R1. | | |
|---|---|---|---|---|---|---|---|
| Spontaneous mammary carcinoma ($C_3H$) | 6 | 7·2 | 2·8 | | 0·43 | 0·54 | |
| | 1 | 16·9 | 5·2 | | | | |
| Spontaneous mammary carcinoma (DBa) | 6 | 16·1 | 3·9 | | 0·70 | 0·60 | |
| | 1 | 23·0 | 6·5 | | | | |
| T 49985—mammary carcinoma* | 6 | 3·3 | 3·7 | 3·9 | 0·47 | 0·44 | 0·47 |
| | 1 | 7·1 | 8·4 | 8·2 | | | |
| | 6 | 3·7 | 2·6 | 2·4 | 0·42 | 0·37 | 0·32 |
| | 1 | 8·7 | 6·8 | 7·4 | | | |
| T 1014—fibro adenoma mamma rat | 6 | 1·8 | 2·8 | | 0·51 | 0·52 | |
| | 1 | 3·5 | 5·4 | | | | |
| Fibro sarcoma mamma (human) | 6 | 0·1 | — | | 0·14 | — | |
| | 1 | 0·7 | — | | | | |
| ,, ,, sclerotic part | 6 | 0·2 | — | | 0·40 | — | |
| | 1 | 0·5 | — | | | | |
| T 86157—lymphosarcoma | 6 | 4 | 4·1 | | 0·23 | 0·24 | |
| | 1 | 17·5 | 16·7 | | | | |
| T 26473—hepatoma. | 6 | 0·86 | 0·92 | 1·8 | 0·36 | 0·35 | 0·49 |
| | 1 | 2·4 | 2·6 | 3·7 | | | |
| Spontaneous hepatoma† " early stage " | 6 | 0·27 | 0·44 | | 0·37 | 0·46 | |
| | 1 | 0·73 | 0·95 | | | | |
| " Fully developed " | 6 | 0·37 | 0·11 | | 0·31 | 0·25 | |
| | 1 | 1·18 | 0·45 | | | | |
| T 5358—interstitial cell carcinoma testis | 6 | 4·5 | 4·4 | | 0·68 | 0·86 | |
| | 1 | 6·7 | 5·1 | | | | |
| T 5441—granulosa cell tumour ovary | 6 | 1·8 | 2·6 | | 0·69 | 0·70 | |
| | 1 | 2·6 | 3·7 | | | | |
| T 26567—sarcomatoid ovarian tumour | 6 | 5·7 | 5·1 | | 0·56 | 0·49 | |
| | 1 | 10·1 | 10·3 | | | | |
| UV 256—sarcoma | 6 | 4·6 | 3·1 | 2·7 | 0·68 | 0·65 | 0·43 |
| | 1 | 6·7 | 4·8 | 6·3 | | | |
| T 17572—adrenal cortex carcinoma | 6 | 2·3 | 3·2 | | 0·60 | 0·72 | |
| | 1 | 3·7 | 4·4 | | | | |

* Tumour transplanted from a spontaneous mammary carcinoma of a DBa female.

† Obtained from 2-year-old female ($C_{57}$ Black × $C_3He$) hybrids ; early stage : small nodules, fully developed hepatomas weighed from 2–10 gm.

One of the smallest R6 : R1 ratios was found in experiments with the lymphosarcoma T 86157. Since the results of the tracer experiments may be influenced by the time during which incubation is carried out, an expectation which stems from the very nature of this experimental procedure employed, the effect of the time of incubation on the R6 : R1 and the Ru : R1 ratios has been studied. The results of such an experiment, using T 86157, are illustrated in Table II ; the specific activities (S.A.) of the barium carbonate prepared from the respiratory $^{14}CO_2$ which was collected during 0·5, 1, 2 and 3 hours are given in Fig. 1. The

FIG. 1.—Relationship between time of incubation and specific activities of $BaCO_3$ prepared from the $^{14}CO_2$ produced from G-1-$^{14}$C, G-u-$^{14}$C and G-6-$^{14}$C by slices of the transplanted lymphosarcoma T86157. Nineteen small-sized tumours were sliced and from the resulting pooled material twelve portions of 1 g. of slices were each incubated with 3 mg. labelled glucose ($5·5 \times 10^3$ c.p.m.).

○——○ G-6-$^{14}$C.

□——□ G-u-$^{14}$C.

▲——▲ G-1-$^{14}$C.

S.A. of the $^{14}CO_2$ from G-1-$^{14}$C was initially much higher than that from G-6-$^{14}$C (1726 against 436 counts per minute after 30 minutes' incubation) but with longer times of incubation the curve representing the S.A. of the latter rose more sharply than that of the former (to 2087 and 1168 counts per minute after 3 hours, respectively). Both the initial level of radioactivity in the $^{14}CO_2$ and the time course of $^{14}CO_2$ production in the two cases were consistent with the theoretical expectation assuming that a direct pathway of glucose oxidation such as that formulated for the HMP " shunt " should be operative in addition to the citric acid cycle. The ratios R6 : R1 and Ru : R1 will thus tend to higher values on increasing the time of incubation.

TABLE II.—*Effect of Time on the Incorporation of Carbon Atoms* 1 *and* 6 *from Specifically Labelled Glucose into Carbon Dioxide by Slices of the Lymphosarcoma T* 86157.

1 g. of slices was incubated with 3 mg. $^{14}C$-glucose at 37° C.

| From : | | Radioactivity recovered in $^{14}CO_2$ (per cent) | | |
|---|---|---|---|---|
| | After : 0·5 hour. | 1 hour. | 2 hours. | 3 hours. |
| G-6-$^{14}C$ . . . | 1·39 | 3·55 | 8·50 | 13·8 |
| G-1-$^{14}C$ . . . | 6·59 | 12·3 | 17·3 | 24·7 |
| G-u-$^{14}C$ . . . | 3·88 | 7·71 | 13·8 | 20·6 |
| Ru : R1 . . . | 0·59 | 0·63 | 0·79 | 0·83 |
| R6 : R1 . . . | 0·21 | 0·35 | 0·49 | 0·56 |

Table III illustrates the results of a similar experiment performed with slices of rat heart. In this case the S.A. of the $^{14}CO_2$ from both G-1-$^{14}C$ and G-6-$^{14}C$ rise concurrently with time. Although small differences in the R6 : R1 ratios with time were consistently observed in the latter experiments, the data did not permit the conclusion that the HMP oxidative pathway was present. The latter might perhaps be made apparent if the citric acid cycle oxidations were blocked by adding an inhibitor such as malonate, because such a procedure should result in a situation somewhat resembling very short times of incubation. Addition of malonate to study the conversion of labelled glucose in liver was applied by Agranoff, Colodzin and Brady (1954*b*).

TABLE III.—*Effect of Time on the Incorporation of Carbon Atoms* 1 *and* 6 *of Specifically Labelled Glucose into Carbon Dioxide by Slices of Rat Heart.*

1 g. of slices were incubated with 3 mg. $^{14}C$-glucose (see Methods : specific activity $5·5 \times 10^3$ c.p.m.) at 37° C.

| Time (hours). | G-n-$^{14}C$ (n). | $BaCO_3$ (mg.). | $BaCO_3$ (c.p.m.). | Rn (per cent). | R6/R1. |
|---|---|---|---|---|---|
| 1 | 6 | 10·1 | 740 | 6·79 | 0·93 |
| | 1 | 10·0 | 810 | 7·36 | |
| 2 | 6 | 12·9 | 1174 | 13·8 | 0·99 |
| | 1 | 12·9 | 1200 | 14·1 | |
| 3 | 6 | 18·2 | 1694 | 28·0 | 1·05 |
| | 1 | 17·9 | 1694 | 26·7 | |

(b) *Effect of malonate on the* $^{14}C$ *recoveries in carbon dioxide*

In the case of a tissue in which the HMP oxidative pathway was completely absent, there should be no difference in the S.A. of, or in the total recovery of isotope in, the $^{14}CO_2$ derived from G-1-$^{14}C$ and G-6-$^{14}C$ ; the addition of malonate would here be expected to cause a lowering of both S.A.'s to the same extent. If, on the other hand, the fall in the S.A. of the $^{14}CO_2$ from G-1-$^{14}C$ as a result of malonate addition should be less than that observed for the $^{14}CO_2$ from G-6-$^{14}C$, this would mean that the extra-glycolytic pathway had revealed itself in consequence of the inhibition of the citric acid cycle.

The latter was actually found true with several normal rat tissues such as heart, kidney, brain and diaphragm, which yielded R6 ; R1 ratios of approximately unity when incubated in the absence of malonate for various periods. When malonate (0·03 M) was added, the R6 : R1 ratios were significantly smaller than unity as can be seen from typical examples listed in Table IV.

TABLE IV.—*Effect of Malonate on the Incorporation of Carbon Atoms* 1 *and* 6 *from Specifically Labelled Glucose into Carbon Dioxide by Slices of Normal Rat Tissues.*

Incubation with 3 mg. of $^{14}C$-glucose (specific activity $5 \cdot 5 \times 10^3$ c.p.m.). Slices of the organs from several rats were pooled before transfer to the four incubation flasks of each experiment. Malonate 0·03 M.

| Tissue mg. per flask. | Incubation period (hours). | Malonate absent. $Ba^{14}CO_3$ from G-n-$^{14}C$ counts per minute. n=6. | n=1. | $^{14}C$-recovery (per cent) as R6/R1. | Malonate present. $Ba^{14}CO_3$ from G-n-$^{14}C$ counts per minute. n=6. | n=1. | $^{14}C$-recovery (per cent) as R6/R1. |
|---|---|---|---|---|---|---|---|
| Heart (650) | 3 | 1794 | 1831 | 20·5/19·6=1·05 | 357 | 808 | 1·56/3·76=0·42 |
| Kidney (1000) | 3 | 1393 | 1581 | 80·0/79·0=1·01 | 372 | 663 | 8·48/16·4=0·52 |
| Diaphragm (600) | 2 | 979 | 1005 | 6·59/6·76=0·98 | 370 | 480 | 1·28/1·71=0·76 |
| Brain (1000) | 1 | 1428 | 1365 | 19·6/18·1=1·08 | 262 | 597 | 1·76/3·79=0·46 |
| | | n=u. | | Ru. | n=u. | | Ru. |
| Diaphragm (1000) | 1 | 586 | | 2·1 | 600 | | 0·6 |
| Brain (1000) | 1 | 2730 | | 24·0 | 2036 | | 9·8 |
| | 2 | 2782 | | 39·2 | 1919 | | 12·4 |
| Heart (500) | 1 | 1045 | | 5·5 | 528 | | 1·3 |
| Kidney (500) | 1 | 1700 | | 45 | 1300 | | 20 |

It may thus be concluded that these tissues are dependent upon the citric acid cycle for oxidation of carbon atom 1 of glucose and that the HMP oxidative pathway does not appear to be operative to such an extent as to be demonstrable by the tracer experiments. By inhibiting the cycle, however, the HMP oxidative pathway becomes manifest, although, as judged from the total amount of isotope recovered, the latter can only account for a small fraction of the $C_1$ atoms of glucose normally oxidized.

When the S.A.'s of the $^{14}CO_2$ produced from G-u-$^{14}C$, with or without malonate present, were compared, in general no significant fall was noted (Table IV, lower part). Since the quantity of $BaCO_3$ recovered in the presence of the citric acid cycle block was always less, the total amount of isotope recovered in the $^{14}CO_2$ was also markedly less than that found in the absence of malonate. The fact that the S.A. of the $^{14}CO_2$ originating from G-u-$^{14}C$ remained of the same order despite the presence of malonate, might have been due to the decarboxylation of the uniformly labelled pyruvate yielding acetylcoenzyme A (followed by formation of acetoacetate). Perhaps a recycling of the labelled substrate through the pentose cycle (de la Haba, Leder and Racker, 1955) must also be kept in mind.

Next, a number of tumours and lung tissue, in which the HMP oxidative pathway had been found to be operative,* were studied in the presence of malonate.

* Rat liver (R6 : R1 = 0·20) and spleen (R6 : R1 = 0·60) belong to this type of tissue also.

The S.A. of the $^{14}CO_2$ produced from G-1-$^{14}$C may be expected to rise under the latter condition, as compared with that collected in the absence of malonate, since the isotope " dilution " by way of the citric acid cycle is prohibited. On the other hand, the $^{14}$C-content of the carbon dioxide, originating from G-6-$^{14}$C, will fall, the citric acid cycle in this case being the only route by which radioactive $CO_2$ can be produced.

The data of Table V show that the latter is invariably found to be true. In the presence of malonate, practically no radioactivity appeared in the carbon dioxide which was produced from G-6-$^{14}$C by slices of the sarcoma UV 256, the lymphosarcoma T 86157 and the ovarian granulosa cell tumour T 5441, indicating that the citric acid cycle oxidations were almost completely blocked. The carbon dioxide formed in the presence of malonate from G-6-$^{14}$C by slices of the testicular tumours (T 5358 and T 26554) and the spontaneous mammary carcinomas of DBa female mice contained more $^{14}$C than the $CO_2$ produced by the slices of the former group of tumours. This suggests that in the latter tumours oxidation through the citric acid cycle had still taken place, although on a much smaller scale than in the absence of malonate, as judged from the total amount of isotope recovered.

The S.A. of the $^{14}CO_2$ from G-1-$^{14}$C in the presence of malonate was higher than in its absence in experiments with four of the tumours (T 5358, UV 256, T 86157 and T 5441) ; this was not the case with two other tumours (the mammary carcinoma and T 26554) and normal lung tissue. However, the S.A. of the $^{14}CO_2$ formed from G-1-$^{14}$C in the presence of malonate by the latter three tissues never

TABLE V.—*Effect of Malonate on the Incorporation of Carbon Atoms* 1 *and* 6 *from Specifically Labelled Glucose into Carbon Dioxide by Slices of Transplanted Mouse Tumours and Lung Tissue.*

1 g. of slices was incubated for 1 hour at 37° C. with 3 mg. $^{14}$C-glucose (specific activity $5 \cdot 5 \times 10^3$ c.p.m.).

| | Malonate absent. | | | Malonate present. | | |
|---|---|---|---|---|---|---|
| | $Ba^{14}CO_3$ from G-n-$^{14}$C counts per minute. | | $^{14}$C-recovery (per cent) as R6/R1. | $Ba^{14}CO_3$ from G-n-$^{14}$C counts per minute. | | $^{14}$C-recovery (per cent) as R6/R1. |
| Tissue. | n=6. | n=1. | | n=6. | n=1. | |
| T 5358 | 887 | 1629 | 6·0/11·7=0·51 | 296 | 2760 | 1·3/10·0=0·13 |
| ,, | 594 | 1234 | 3·8/7·9=0·48 | 156 | 1904 | 0·8/9·3=0·09 |
| T 26554 | 1236 | 1766 | 8·6/11·9=0·72 | 376 | 1513 | 2·3/9·2=0·25 |
| Spontaneous mammary carcinoma (DBa) | 814 | 1493 | 3·94/6·5=0·60 | 285 | 1229 | 0·99/5·46=0·18 |
| UV 256 | 628 | 1282 | 4·0/6·9=0·58 | 35 | 1389 | 0·1/5·0=0·02 |
| T 86157 | 470 | 2365 | 2·6/14·6=0·18 | 39 | 3452 | 0·07/8·5=0·008 |
| ,, * | 506 | 2794 | 2·8/11·7=0·21 | 69 | 3535 | 0·2/11·8=0·01 |
| T 5441 | 493 | 1071 | 1·3/3·3=0·40 | 0 | 1397 | 0/2·0=0 |
| Lung† | 1700 | 2977 | 27·7/46·5=0·57 | 485 | 2217 | 6·26/25·0=0·25 |

| | n=u. | Ru. | n=u. | Ru. |
|---|---|---|---|---|
| T 86157* | 1433 | 6·1 | 2116 | 6·3 |
| T 5441 | 1222 | 5·8 | 1576 | 4·9 |
| T 5358 | 1440 | 8·0 | 1521 | 6·8 |

* Same experiment.

† Incubated during 3 hours.

showed such a drop as was found when the tissues listed in Table IV were incubated in the presence of malonate.

Consequently, the total recovery of isotope in the $^{14}CO_2$ produced from G-1-$^{14}$C was not at all or only slightly diminished following the inhibition of the citric acid cycle of all the tumours studied. The same was found for G-u-$^{14}$C.

This stands in contrast to what was found with the normal tissues listed in Table IV, which were much more dependent upon the citric acid cycle for oxidation. In comparing these results it should also be noted that in experiments with the latter tissues, malonate reduced the quantity of $CO_2$ (recovered as mg. $BaCO_3$) to a greater extent than in the experiments with the tumours.

(c) *Effect of monoiodoacetate (MIA) on the $^{14}C$ recoveries in carbon dioxide*

Dickens and Glock (1951) have shown that iodoacetamide in a concentration of 0·01 M had very little effect on the activities of the glucose 6–phosphate and 6-phosphogluconate dehydrogenases of liver. The formation of sedoheptulose, an intermediate of the HMP oxidative pathway, from glucose 6-phosphate and triphosphopyridine nucleotide was not affected in the presence of MIA ($5 \cdot 4 \times 10^{-4}$M) in our experiments with soluble enzyme preparations from the tumours (Bosch, van Vals and Emmelot, 1956). The formation of sedoheptulose from glucose in tumour slices incubated in the presence or absence of MIA could not be demonstrated by spectrophotometric means. In the presence of MIA, hexose concentration fell off very slowly and triose accumulated as shown by the anthrone reaction ; in addition the formation of fructose could be demonstrated by means of the cysteine-sulfuric acid method (Bosch, van Vals and Emmelot, 1956). Although no exact quantitative measurements of the intermediates were made, it was nevertheless apparent from the absorption spectra obtained that glucose disappearance was very markedly less than in the absence of MIA. In the presence of MIA, lactic acid formation was also significantly diminished.

As a result of the glycolytic block exerted by MIA, the amount of isotope recovered in the carbon dioxide which was produced from G-u-$^{14}$C was diminished ; for example, 500 mg. of slices of the testis tumour T 5358 produced 3·1 mg. $BaCO_3$ of 875 counts/min. but in the presence of MIA only 2·6 mg. $BaCO_3$ of 345 counts/min. If the Embden-Meyerhof pathway had been blocked completely at the triose phosphate stage by MIA, any $^{14}CO_2$ recovered should have been formed exclusively by glucose oxidation via the HMP oxidative pathway. However, our experiments are not conclusive with regard to the effectiveness of the MIA block. The experiments with G-u-$^{14}$C demonstrated that in the presence of MIA relatively more unlabelled than labelled substrate became oxidized as compared with oxidation in the absence of the inhibitor. This is easily understood since the block slows down glucose dissimilation, but still allows endogenous compounds, such as lactic acid and fatty acids to be oxidized through the citric acid cycle. No radioactivity would be present in the $^{14}CO_2$ produced from G-6-$^{14}$C if the glycolytic pathway was completely blocked in the presence of MIA. It was found, however, that the S.A's of the $^{14}CO_2$ from both G-6-$^{14}$C and G-1-$^{14}$C were diminished to about half of the original value. Accordingly the R6 : R1 ratios did not differ much either in the presence or absence of MIA. Thus, either the glycolytic block had been inefficient or a randomization of the tracer from G-6-$^{14}$C had occurred as a result of which $^{14}CO_2$ was produced by a pathway other than the citric acid cycle. Since triose accumulates as a result of the inhibition by MIA, an equilibration of tracer via glyceraldehyde 3-phosphate ⇌ dioxyacetone phosphate might easily have taken place. A resynthesis to hexose would then mean that carbon atom 6 of the original glucose had been converted into carbon atom 1 of the newly synthesized hexose. This phenomenon would tend to dilute the S.A. of the $^{14}CO_2$ produced from G-1-$^{14}$C and in the case of G-6-$^{14}$C would produce radioactive carbon dioxide through the HMP oxidative pathway. As regards the other possibility of inefficient blocking, when the concentration of MIA was raised ten-fold, the S.A. of the carbon dioxide obtained from only some of the preliminary experiments with G-6-$^{14}$C, still remained of the same order as the corresponding values of the earlier experiments. Further investigations are being carried out in this connection.

### SUMMARY

Normal and neoplastic tissues were incubated with uniformly labelled glucose and glucose specifically labelled in either carbon atom 1 or carbon atom 6. From the recovery of isotope in the carbon dioxide produced, it followed that in all neoplastic tissues studied a marked difference in the rates of metabolism between carbon atom 1 and 6 did exist.

Furthermore, observations on the effect of the time of incubation and of the presence of a citric acid cycle block (malonate) were consistent with the view that the hexose monophosphate oxidative pathway was involved in the glucose metabolism of the tumours. In some experiments the effect of a glycolytic block (monoiodoacetate) was also studied. In a number of normal rat tissues (heart, kidney, brain, diaphragm) the existence of the hexose monophosphate oxidative pathway was only revealed when incubation was carried out in the presence of malonate.

### REFERENCES

Abraham, S., Cady, P. and Chaikoff, I. L.—(1956) *Proc. Amer. Ass. Cancer Res.*, **2**, 89.
*Idem*, Hill, R. and Chaikoff, I. L.—(1955) *Cancer Res.*, **15**, 177.
*Idem*, Hirsch, P. F. and Chaikoff, I. L.—(1954) *J. biol Chem.*, **211**, 31.
Agranoff, B. W., Brady, R. O. and Colodzin, M.—(1954) *Ibid.*, **211**, 773.
*Idem*, Colodzin, M. and Brady, R. O.—(1954) *Fed. Proc.*, **13**, 173.
Barron, E. S. G., Villavicencio, M. and King, Jr., D. W.—(1955) *Arch. Biochem.* **58**, 500.
Bloom, B. and Stetten, Jr., D.—(1953) *J. Amer. chem. Soc.*, **75**, 5446.
Bosch, L., van Vals, G. H. and Emmelot, P.—(1956) *Brit. J. Cancer*, **10**, 2nd paper.
Dickens, F.—(1956) *Third int. Congr. Biochem.*, Brussels, 1955. (C. Liébecq, ed.) New York (Academic Press), p. 170.
*Idem* and Glock, G. E.—(1951) *Biochem. J.*, **50**, 81.
Emmelot, P., Bosch, L. and van Vals, G. H.—(1955) *Biochim. Biophys. Acta*, **17**, 451.
Glock, G. E. and McLean, P.—(1954) *Biochem. J.*, **56**, 171.
de la Haba, G., Leder, I. G. and Racker, E..—(1955) *J, biol. Chem.*, **214**, 409.
Kit, S.—(1956) *Cancer Res.*, **16**, 70.
*Idem* and Graham, O. L.—(1956) *Ibid.*, **16**, 117.
Lewis, K. F., Blumenthal, H. J., Wenner, C. E. and Weinhouse, S.—(1954) *Fed. Proc.*, **13**, 252.
Racker, E.—(1954) *Advanc. Enzymol.*, **15**, 141.
Wenner, C. E., Bloch-Frankenthal, L. and Weinhouse, S.—(1956) *Proc. Amer. Ass. Cancer Res.*, **2**, 156.